# Zwischen Bagatellisierung und Pathologisierung: Gesundheitsversorgung im Alter und die Zeitstruktur guten Lebens

**DOI:** 10.1007/s00481-022-00742-6

**Published:** 2023-01-04

**Authors:** Mark Schweda, Eva Hummers, Evelyn Kleinert

**Affiliations:** 1 Abteilung Ethik in der Medizin, Department für Versorgungsforschung, Carl von Ossietzky Universität Oldenburg, Ammerländer Heerstr. 114–118, 26129 Oldenburg, Deutschland; 2 Institut für Allgemeinmedizin, Universitätsmedizin Göttingen, Göttingen, Deutschland

**Keywords:** Altern, Gutes Leben, Gesundheitsversorgung, Zeit, Strebensethik, Ageing, Good life, Healthcare, Temporality, Teleological ethics

## Abstract

Steigende Lebenserwartung, sozialer Wandel und medizinische Innovationen fordern traditionelle Sichtweisen auf das Alter(n) heraus. Was einst als eine „normale“ Alterserscheinung galt, wird heute im Lichte veränderter Lebensentwürfe und neuartiger Interventionsmöglichkeiten oft schon als Erkrankung aufgefasst und behandelt. Altersbezogene Gesundheitsstandards und Behandlungsziele geraten in Bewegung. Es eröffnet sich ein Spannungsfeld zwischen Bagatellisierung und Pathologisierung von Alterungsprozessen, das der ethischen Reflexion bedarf. Der Beitrag geht der Frage nach, wie individuelle und gesellschaftliche Vorstellungen des Alter(n)s im Kontext der modernen Medizin ethisch zu verstehen sind. Dazu geben wir zunächst einen Überblick zur Rolle von Altersbildern in der medizinischen und pflegerischen Versorgung älterer Menschen. Anschließend werden begrifflich-theoretische Perspektiven umrissen, die solche Bilder des Alter(n)s einer strebensethischen Analyse zugänglich machen. Welche Formen der Gesundheitsversorgung im höheren Alter als sinnvoll und angemessen zu gelten haben, ist demnach nicht zuletzt im Licht der Frage nach der Zeitstruktur guten Lebens zu diskutieren.

## Abstract (en)

**Definition of problem:**

Modern medicine is challenging traditional views of age(ing). What was long considered a “normal” sign of old age is now often perceived and treated as a disease. As a result, age-related health standards and treatment goals are shifting. The resulting scope between trivialization and pathologization of age(ing) requires ethical reflection.

**Argument:**

This article explores the question of how notions of age(ing) are to be understood ethically in the context of medicine. We first provide an overview of the state of research on the role of age stereotypes in the healthcare of older people. The notions of age(ing) identified in this context are then analyzed from the perspective of teleological ethics.

**Conclusions:**

What kinds of healthcare are reasonable and appropriate in old age has to be discussed in the context of the temporal structure of a good life.

## Einleitung

„Wir retten möglicherweise Menschen, die in einem halben Jahr sowieso tot wären“, erklärte Boris Palmer mit Blick auf die weitreichenden Maßnahmen, die zu Beginn der COVID-19-Pandemie zum Schutz älterer Menschen ergriffen wurden. „Wenn Sie die Todeszahlen durch Corona anschauen“, fügte der Tübinger Oberbürgermeister hinzu, „dann ist es […] so, dass viele Menschen über 80 sterben – und wir wissen, über 80 sterben die meisten irgendwann“ (Tagesspiegel 2020).

Die Auffassung, dass ältere Menschen unweigerlich dem Tod entgegengehen, sodass medizinisch nicht mehr viel für sie zu tun sei, hat Tradition. Das Nachlassen von Gesundheit, Lebensqualität und Leistungsfähigkeit im höheren Lebensalter galt lange als naturgegebener, schicksalhafter Verlauf des menschlichen Lebens. Altern und Alter erschienen entsprechend als äußerste Grenze ärztlicher Kunst und medizinischer Zuständigkeit (Schäfer 2004). Mit der Entwicklung der Altersmedizin im 20. Jahrhundert verlor diese Haltung an Boden. Sie führte die grundlegende Unterscheidung von physiologischem Altern und Alterserkrankungen ein, die medizinisch zu behandeln sind. Von nun an bedeutete Altwerden nicht mehr notwendig Krankheit und Gebrechlichkeit. Die Medizin erhob den Anspruch, einen maßgeblichen Beitrag zu einem gesunden und funktionsfähigen Leben im höheren Alter leisten zu können (Schäfer 2004).

Inzwischen rückt das Altern zunehmend ins Blickfeld medizinischer Anstrengungen. Die Vorstellung eines natürlichen Versiegens der Lebenskräfte im Zuge des Älterwerdens erscheint im Licht wissenschaftlicher Erklärungsmodelle und technischer Eingriffsmöglichkeiten oft nicht mehr akzeptabel. Die Kategorie der Altersschwäche ist aus Totenscheinen und Sterbestatistiken verschwunden. Altersassoziierte Seneszenzprozesse sollen ätiologisch aufgeschlüsselt und kausal behandelt werden (Schweda 2017a). Die so genannte Anti-Aging-Medizin scheint daraus nur den konsequenten Schluss zu ziehen, mit der fortschreitenden Eliminierung der Ursachen aller gesundheitlicher Beeinträchtigungen des höheren Lebensalters müsste sich schließlich auch das, was wir heute noch als unausweichliches Altern ansehen, vollends verflüchtigen (Schweda 2017a). Ihre Vorreiter erklären, der medizinische Fortschritt ermögliche „die Eliminierung sämtlicher Behinderungen, Deformationen, Schmerzen, Krankheiten, Leiden und Sorgen des Alters“ (Klatz und Goldman 2003, S. 13).

Vor diesem Hintergrund geht der folgende Beitrag der ethischen Bedeutung individueller und gesellschaftlicher Bilder des Alterns und ihres Wandels im Horizont von Medizin und Gesundheitsversorgung nach. Zunächst wird anhand ausgewählter sozialwissenschaftlicher Studien schlaglichtartig beleuchtet, wie altersbezogene Normen medizinischer Behandlung und pflegerischer Unterstützung in Bewegung geraten und sich ein Spannungsfeld zwischen Bagatellisierung und Pathologisierung von Alterungsprozessen eröffnet. Im Anschluss werden begrifflich-theoretische Perspektiven umrissen, die die zu Grunde liegenden Altersbilder einer strebensethischen Analyse zugänglich machen. Welche Formen der Gesundheitsversorgung im höheren Alter als sinnvoll und angemessen zu gelten haben, ist demnach nicht zuletzt im Licht der Frage nach der zeitlichen Struktur eines guten Lebens zu diskutieren. Abschließend werden Potenziale und Herausforderungen einer solchen Betrachtungsweise aufgezeigt und theoretisch-methodologische Hinweise für die Auseinandersetzung mit ihnen formuliert.

## Zur Bedeutung von Altersbildern in Medizin und Gesundheitsversorgung

Altersbilder sind Vorstellungen vom Altern als Prozess, dem höheren Alter als Lebensphase sowie älteren Menschen als Gruppe (BMFSFJ 2010; Rossow 2012, S. 11). Sie können vielfältige Auswirkungen haben, sich etwa zu sozialen Stereotypen verfestigen und altersbezogene Stigmatisierung sowie Diskriminierung (Ageism) begünstigen (Berner et al. 2012). Auch im Kontext der Gesundheitsversorgung, die hier vorrangig den Bereich medizinischer Behandlung und pflegerischer Unterstützung umfasst, spielen solche Vorstellungen eine nicht zu unterschätzende Rolle (Berner et al. 2012, S. 157–368). Sie sind insbesondere von Bedeutung für das Gesundheits- und Inanspruchnahmeverhalten von Patientinnen und Patienten sowie die Entscheidungen von medizinischem und pflegerischem Fachpersonal.

### Zur Bedeutung von Altersbildern bei Patientinnen und Patienten

Der Zusammenhang von Altersbildern und Gesundheit ist Gegenstand intensiver gerontologischer Forschung (für einen Überblick Wurm 2020). Auch Menschen im höheren Alter achten in Abhängigkeit von Geschlecht, Bildungsstand und Einkommen heute vermehrt auf sportliche Aktivität, gesunde Ernährung und Vorsorgeuntersuchungen (Spuling et al. 2017b; Huy und Thiel 2009). Dabei korrelieren positive Altersbilder mit förderlichem Gesundheitsverhalten und umgekehrt.

Zudem spielen so genannte Kontrollüberzeugungen eine Rolle: Personen, die an einen großen Einfluss des eigenen Verhaltens auf ihre Gesundheit und ihre Lebensbedingungen glauben, stellen eher die Vorteile und den Gewinn an Lebenserfahrung in den Vordergrund. Dagegen nehmen Menschen, die sich dem Schicksal oder gesellschaftlichen Verhältnissen ausgeliefert fühlen, vornehmlich altersbedingte Einschränkungen wahr (Wurm und Tesch-Römer 2006). Schließlich hat auch die Ursachenzuschreibung von Beeinträchtigungen eine wichtige Bedeutung. Studien belegen, dass Menschen, die gesundheitliche Beschwerden ihrem Alter zuschreiben, seltener ärztliche Hilfe suchen als jene, die sie als krankheitsbedingt erachten (Wurm 2020).

Der aktuelle sowie der erwartete Gesundheitszustand haben einen bedeutenden Einfluss auf den Wunsch nach einem möglichst langen Leben (Lang und Rupprecht 2019). Allerdings ist seine Einschätzung sehr subjektiv und unterscheidet sich von objektiven Bewertungen im Sinne ärztlicher Diagnosen (Spuling et al. 2017a). Eine gute subjektive Gesundheit kann auch ein Grund für die Ablehnung einer medizinischen Behandlung oder pflegerischen Unterstützung sein, z. B. bei Patientinnen und Patienten mit verminderter Herzleistung ohne spürbare Symptome, die auf ein elektronisches Implantat verzichten (Ottenberg et al. 2014). Weitere Argumente sind der Glaube an Schicksal, negativ ausfallende Nutzen-Risiko-Abwägungen oder der Wunsch, selbst zu entscheiden, sowie fehlendes Vertrauen in die Medizin (Johnson et al. 2020; Ottenberg et al. 2014). Studien zu gesundheitlichen Vorausverfügungen deuten darauf hin, dass höheres Lebensalter häufiger mit Wünschen nach Unterlassung oder Beendigung von lebensverlängernden Maßnahmen einhergeht (Hug et al. 2020; Block et al. 2019).

### Zur Bedeutung von Altersbildern Versorgender

Altersbilder ärztlicher und pflegerischer Fachkräfte fallen aufgrund der beruflichen Erfahrung mit alten Menschen oft differenzierter aus (Remmers und Walter 2012). Auch bei Fachkräften bestehen positive Altersbilder im Sinne von Reife, Gelassenheit und Lebensfreude. Der Blick auf die (körperliche) Gesundheit wird jedoch weiterhin von eher negativen Vorstellungen geprägt, besonders bei Pflegekräften (Remmers und Renneke 2012). Grundlegend orientieren sich Fachkräfte kaum am kalendarischen Alter, sondern vorwiegend am biologischen.

Eine Herausforderung bei der Behandlung Hochaltriger stellt das gleichzeitige Auftreten mehrerer (chronischer) Erkrankungen (Multimorbidität) dar, da nicht mehr jede leitliniengerechte Therapie mit ihren Risiken und Nebenwirkungen zumutbar erscheint und unter Umständen mehr schadet als nutzt (Søreide und Desserud 2015; Günster et al. 2012). Eine geriatrisch-allgemeinmedizinische Behandlung richtet die Beantwortung der Frage, welche Erkrankungen oder Beeinträchtigungen in welchem Umfang und mit welchen Mitteln behandelt werden sollten, daher an dem Ziel aus, die Autonomie und Lebensqualität älterer Menschen so weit wie möglich zu erhalten und Pflegebedarf zu vermindern (Günster et al. 2012).

Auch sozialempirische Untersuchungen deuten darauf hin, dass die Berücksichtigung des Alters bei der Entscheidungsfindung in der direkten Arzt-Patienten-Beziehung als sachlich angemessen angesehen wird, während chronologisches Alter allein als generelles Entscheidungskriterium, insbesondere für Ressourcenverteilung, abgelehnt wird (Ubachs-Moust 2011). Vereinzelt wird allerdings auch argumentiert, dass negative Altersbilder bei pflegerischem und medizinischem Personal zu einer schlechteren medizinisch-pflegerischen Versorgung alter Menschen führen (Wyman et al. 2018) oder dass negative Altersstereotype diskriminierendes Verhalten fördern können, welches wiederum die psychische und physische Gesundheit von Patientinnen und Patienten negativ beeinflusst (Schroyen et al. 2014).

Wenn Ärzte bzw. Ärztinnen eine medizinische Behandlung als vergeblich einschätzen, begründen sie dies meist damit, dass die Belastungen der Behandlung den Nutzen deutlich übersteigen oder die Ziele des Patienten bzw. der Patientin mit der Behandlung nicht erreicht werden können. Ebenso werden medizinische Interventionen als vergeblich empfunden, wenn der Tod unmittelbar bevorsteht oder absehbar ist, dass ein Überleben außerhalb der Intensivstation nicht mehr möglich ist (Huynh et al. 2013). Aus den Pflegewissenschaften ist bekannt, dass die Arbeitszufriedenheit der Pflegenden sinkt und ihre emotionale Erschöpfung steigt, wenn sie aussichtslose Maßnahmen durchführen müssen bzw. mit der Pflege der so behandelten Erkrankten betraut sind (Borhani et al. 2015).

### Der Wandel von Altersbildern in Medizin und Gesundheitsversorgung

Wie neue medizinische Möglichkeiten Standards altersgemäßer Behandlung verändern, wird etwa am Beispiel koronarer Bypass-Techniken deutlich. Hier wurde *höheres Alter* als Ausschlusskriterium bzw. Risikofaktor ersetzt durch *Vorerkrankungen und Gebrechlichkeit* (Neuman und Bosk 2013). Zugleich traten an die Stelle von Bypässen minimalinvasive Prozeduren, die für noch ältere Menschen geeignet sind. Dies spiegelt sich in Statistiken, wonach Herzoperationen von 2005 bis 2019 insgesamt um das 1,3-Fache, bei über 95-Jährigen aber um das Doppelte zunahmen (BfArM 2022). Der Einsatz externer Defibrillation des Herzrhythmus erhöhte sich bei dieser Gruppe im gleichen Zeitraum sogar um das Fünffache (Statistisches Bundesamt 2021).

Vergleichbare Entwicklungen wurden auch mit Blick auf andere lebensverlängernde kardiologische Eingriffe (Shim et al. 2008) sowie auf Behandlungen wie z. B. Hämodialyse (Russ et al. 2005) und Organtransplantation (Kaufman et al. 2006) bei älteren Menschen verzeichnet. Allgemein werden auf diese Weise immer mehr Maßnahmen auch im fortgeschrittenen Alter zu Standardprozeduren, die sinnvoll, angemessen und notwendig erscheinen. Die Grenzen zwischen normaler Routinebehandlung und außergewöhnlicher medizinischer Intervention geraten in Bewegung und verschieben sich (Kaufman 2015). In der medizinsoziologischen Forschung wird hier auch von einer (Bio‑)Medikalisierung des Alter(n)s gesprochen, also einem Vordringen medizinischer Beschreibungsweisen und Handlungsstrategien mit Blick auf Altern und höheres Lebensalter (Estes und Binney 1989; Kaufman et al. 2004).

Der Verzicht auf eine medizinische Maßnahme aufgrund des vorgerückten Alters der betreffenden Person erscheint unter diesen Vorzeichen nicht mehr ohne Weiteres selbstverständlich und hinnehmbar. Immerhin besteht die Gefahr, dass damit inzwischen gut behandelbare körperliche oder psychische Leiden bzw. Beeinträchtigungen als „normale“ Alterserscheinungen abgetan werden. Eine solche *Bagatellisierung *wäre nicht nur im Hinblick auf die ärztliche Haltung problematisch. Sie könnte auch gravierende praktische Konsequenzen haben, etwa eine unzulängliche Versorgung älterer Menschen (für eine Übersicht Walter und Krugmann 2013). So wurden etwa Symptome psychischer Krankheiten im Alter lange als altersbedingte Veränderungen angesehen und alten Menschen aufgrund negativer Zuschreibungen wie nachlassender psychischer Plastizität oder erschöpfter Entwicklungspotenziale keine psychotherapeutischen Maßnahmen mehr angeboten (Kammerer et al. 2015). Auch ältere Krebspatientinnen und -patienten werden oft nicht leitlinienkonform behandelt und erhalten vielfach eine weniger intensive bzw. extensive onkologische Versorgung (Schroyen et al. 2014). Ähnliches lässt sich ebenfalls mit Blick auf akute Herzerkrankungen feststellen (Schoenenberger et al. 2008).

Andererseits kann eine fortschreitende Medikalisierung des Alterns allerdings auch mit einer *Pathologisierung *von zuvor als „normal“ bzw. „altersgemäß“ angesehenen Erscheinungen einhergehen. Einbußen an Funktionalität und Lebensqualität werden dann etwa unter medizinischen oder pharmakologischen Vorzeichen als Ausdruck krankhafter Veränderungen gedeutet, die der Behandlung bedürfen (Estes und Binney 1989; Kaufman et al. 2004). In der Folge erscheinen Altern und höheres Lebensalter insgesamt zunehmend als eine Zeit vermeidbarer Morbidität und Mortalität, in der „vorausschauend“ und „risikoorientiert“ zu behandeln ist (Shim et al. 2008, S. 344). Eine solche Sichtweise kann nicht nur die individuelle und gesellschaftliche Akzeptanz altersassoziierter körperlicher oder psychisch-mentaler Veränderungen untergraben und damit negative, stigmatisierende Altersbilder verstärken (Kaufman et al. 2004). Gerade im Einzugsbereich der bereits erwähnten Anti-Aging-Medizin droht die Pathologisierung von Alterungsprozessen auch zu Fehlversorgung und gesundheitlichen Risiken zu führen, wenn etwa das Absinken gewisser Hormonspiegel über den Lebensverlauf mit endokrinologischen Mangelerkrankungen in jüngeren Jahren gleichgesetzt und entsprechend durch medizinisch nicht indizierte „Hormon(ersatz)therapien“ behandelt wird (Perls und Handelsman 2015).

## Strebensethische Perspektiven

Wie sich zeigt besteht ein enges Wechselverhältnis zwischen Altersbildern und Normen des medizinisch Sinnvollen, individuell Erstrebenswerten und gesellschaftlich Akzeptablen: Auf der einen Seite kann ein traditionelles Bild des Alterns als Prozess unvermeidlichen körperlichen und geistigen Verfalls eine Bagatellisierung und unzulängliche Versorgung altersassoziierter Beschwerden begünstigen. Auf der anderen droht die Aussicht einer medizinisch optimierten Lebensqualität und Leistungsfähigkeit im Alter einer Pathologisierung vormals als natürlich geltender Begleiterscheinungen des Älterwerdens Vorschub zu leisten. In beiden Fällen scheinen allerdings umfassendere Wertvorstellungen zum Tragen zu kommen, die einer Klärung und Erörterung bedürfen.

Aus einer ethischen Perspektive lassen sich hier ausgehend von der Diskussion um Anti-Aging-Medizin zwei divergierende Bewertungsperspektiven herausarbeiten (Overall 2020; Schweda 2017a; Ehni 2014). Vertreter der einen Seite betrachten das Alter(n) als eine hinzunehmende oder bejahenswerte Gegebenheit. Dabei werden Vergänglichkeit und Endlichkeit als Bedingung eines sinnvollen Lebens und Ausdruck einer natürlichen Ordnung des individuellen und generationellen Lebenszyklus gedeutet (zur Übersicht Eichinger und Bozzaro 2011). Vertreter der anderen Seite sehen das Alter(n) dagegen als Übel und Hindernis eines erfüllten Lebens an. Ihnen zufolge würde seine Überwindung neue Aussichten eröffnen, beglückende Erfahrungen zu genießen, erstrebenswerte Güter zu erlangen oder maßgebliche Wünsche zu erfüllen. Darüber hinaus würde sie mehr Spielraum für die Entwicklung, Erprobung und Verwirklichung unterschiedlichster Vorhaben und Lebensentwürfe schaffen (Eichinger und Bozzaro 2011).

Diese Kontroverse verdeutlicht, wie Fragen von Sinn und Angemessenheit medizinischer Interventionen mit Blick auf das Alter(n) mit strebensethischen Perspektiven eines guten Lebens in der Zeit verschränkt sind. Allerdings konzentriert sie sich vor allem auf extreme Szenarien radikaler Lebensverlängerung. Eine vergleichbare ethische Betrachtung wirklichkeitsnäherer und praktisch relevanterer Ansätze, etwa im Bereich regulärer hausärztlicher oder geriatrischer Versorgung, steht hingegen aus. Entsprechend wird in der Erörterung von Fragen des guten Lebens in diesem Zusammenhang zumeist auch nur der Aspekt der zeitlichen Dauer in den Blick genommen (Knell 2015). Andere wichtige Dimensionen wie die Irreversibilität, die Segmentierung und Verlaufsstruktur des Lebens oder die narrative Strukturierung der individuellen Lebensgeschichte bleiben weitgehend unberücksichtigt (Schweda 2020).

Im Folgenden wird daher ein begrifflich-theoretischer Rahmen entworfen, der eine eingehendere ethische Auseinandersetzung mit Altersbildern im Kontext von Medizin und Gesundheitsversorgung im Hinblick auf die darin vorausgesetzten Vorstellungen guten Lebens in der Zeit ermöglichen soll. Dazu werden zunächst im Ausgang von Ansätzen der philosophischen Ethik theoretische Perspektiven auf ein gutes Leben unterschieden und ihre Implikationen für Medizin und Gesundheitsversorgung im höheren Alter aufgezeigt. Im Anschluss werden diese Perspektiven dann auf drei verschiedene Ebenen menschlicher Zeitlichkeit bezogen, um eine differenziertere Analyse der Bedeutung der zeitlichen Struktur guten Lebens für medizinethische Fragen im höheren Lebensalter zu ermöglichen (dazu vorbereitend Stange und Schweda 2022).

### Perspektiven guten Lebens und Gesundheitsversorgung im Alter

Welche Bedeutung die in Altersbildern implizierten Vorstellungen eines guten Lebens für Medizin und Gesundheitsversorgung im Alter haben können, lässt sich anhand der philosophischen Unterscheidung hedonistischer, subjektiver und objektiver Theorien guten Lebens (Steinfath 1998) exemplarisch verdeutlichen. Dabei werden diese unterschiedlichen Perspektiven im vorliegenden Zusammenhang nicht als einander ausschließend aufgefasst. Sie können jeweils berechtigte, wenn auch mitunter konkurrierende Teilaspekte eines guten Lebens zur Geltung bringen.

Für *hedonistische Theorien* sind Lust und Unlust die maßgeblichen Gesichtspunkte. Ein gutes Leben ist demnach eines, das möglichst viele lustvolle, von Freude, Genuss und Vergnügen, und möglichst wenige von Leid, Schmerz und Verdruss geprägte Erlebnisse beinhaltet (Steinfath 1998). Eine solche Perspektive scheint auch vielen negativen, defizitorientierten Altersbildern zugrunde zu liegen. Demnach schwinden im Alter Freude und Vergnügen, etwa aufgrund körperlicher Einschränkungen, und Schmerzen und Verstimmung nehmen infolge von Krankheit, psychischer Belastung und sozialer Isolation zu (Beyer et al. 2017). Solche Vorstellungen unweigerlich abfallender Lebensqualität spielen in der Versorgungspraxis eine nicht zu unterschätzende Rolle. So setzt schon die ärztliche Einschätzung von Nutzen bzw. Vergeblichkeit medizinischer Maßnahmen im höheren Alter Annahmen über die durch sie zu erzielende Lebensqualität voraus (Schneiderman 1994). In sozialpolitischen Debatten um Ressourcenverteilung wird sogar für eine altersbezogene Begrenzung der Versorgung ins Feld geführt, dass der in qualitätsbereinigten Lebensjahren bemessene Nutzen medizinischer Maßnahmen bei alten Menschen niedriger ausfalle, da hier per se nur geringe Lebensqualitätszuwächse zu erzielen seien (Brock 2003). Freilich lässt sich ein rein hedonistisches Verständnis von Lebensqualität kritisieren. In der philosophischen Tradition wurde etwa argumentiert, dass das Alter(n) bei allen Beschwernissen zugleich von Trieben und Leidenschaften befreie und so erst für das wahrhafte Glück der Freundschaft, der ästhetischen Bewunderung oder der theoretischen Betrachtung empfänglich mache (Anton 2016). In dieser Hinsicht kann eine ethische Reflexion der Bedingungen guten Lebens im Alter dazu beitragen, einseitig negative Altersbilder und Lebensqualitätsurteile in Medizin und Gesundheitsversorgung zu problematisieren.

Für *subjektive Theorien* steht nicht Lust, sondern die Erfüllung zentraler Wünsche im Vordergrund. Ein gutes Leben ist demnach eines, in dem maßgebliche Vorhaben realisiert und Ziele erreicht werden (Steinfath 1998). Aus dieser Sicht erscheint die strebensethische Einschätzung von Medizin und Gesundheitsversorgung im höheren Alter bereits vielschichtiger. So können Schmerz und Leid in Kauf genommen werden, um für das eigene Leben zentrale Wünsche zu verwirklichen, etwa die Beilegung eines lange bestehenden Konflikts oder die Vollendung eines bedeutsamen Vorhabens (Fuchs 2012). Umgekehrt kann selbst ein schmerz- und belastungsfreies oder gar genussreiches Leben jeden Wert verlieren, wenn wesentliche Ziele und Vorhaben nicht mehr zu verwirklichen sind. So rekurrieren etwa Debatten um die Bedeutung eines „vollendeten Lebens“ für Entscheidungen am Lebensende und ärztlich assistierten Suizid im höheren Alter auf die Möglichkeit, die Verwirklichung der leitenden Zielsetzungen des eigenen Lebens gleichsam „zu überleben“ (Gilleard 2020). Letztlich hängen die Aussichten eines guten Alter(n)s aus Sicht von Wunschtheorien vom konkreten Inhalt der jeweiligen lebensbestimmenden Präferenzen ab. Die Verfolgung mancher Wünsche, etwa nach einer bestimmten beruflichen oder familiären Weichenstellung, mag nur schwer mit einem fortgeschrittenen Alter vereinbar sein. Dagegen kann die Verwirklichung anderer Wünsche so viel Zeit in Anspruch nehmen, dass sie überhaupt erst im späteren Leben in Reichweite kommt, etwa der Wunsch nach Enkelkindern oder den Freiheiten des Ruhestands. Der jeweilige Stand der biographischen „Wunscherfüllungsbilanz“ dürfte jedenfalls auch für die Bewertung medizinischer Angebote im höheren Alter von Belang sein. Dabei ist zu berücksichtigen, dass Wünsche über den Lebensverlauf nicht konstant bleiben. Gerade Erfahrungen von Krankheit, Einschränkung und Behinderung können dazu führen, eigene Ziele und Vorhaben zu überdenken und im Licht der gegebenen Möglichkeiten anzupassen (Spuling et al. 2017a). Diese Anpassung der Bewertungsmaßstäbe mag den erstaunlichen Anstieg der subjektiven Lebenszufriedenheit im fortgeschrittenen Lebensalter erklären, die die im mittleren Alter weit übertrifft (Wareham 2022).

Im Rahmen *objektiver Theorien *bemisst sich das Gelingen eines Lebens an Gütern, Werten oder Zwecken, die unabhängig von subjektiven Empfindungen und persönlichen Präferenzen als gut, sinnvoll oder erstrebenswert zu gelten haben (Steinfath 1998). Ungeachtet der kontroversen Frage, wie sich eine derartige objektive Perspektive überhaupt begründen ließe und welche inhaltlichen Bestimmungen sie dem guten Leben gäbe, lassen sich auch hier allgemeine Implikationen für die ethische Einschätzung des Alterns und höheren Alters aufzeigen. Jedenfalls erscheint ein gutes Leben in Abhängigkeit von dem konkreten Set objektiver Güter oder Werte auch in dieser Lebensphase durchaus möglich. Objektive Theorien wie der Befähigungsansatz entfalten etwa auf einer anthropologischen Grundlage eine differenzierte Darstellung wesentlicher menschlicher Befähigungen, deren Entfaltungsmöglichkeit als grundlegende Bedingung eines guten Lebens aufgefasst wird (Nussbaum 2011). Auch wenn bislang keine systematische Explikation dieser Bedingungen mit Blick auf das Altern und höhere Alter vorliegt, können sie doch auch hier von erheblicher Bedeutung sein. Dies gilt gerade für Aspekte der körperlichen Integrität, geistigen Betätigung und des sozialen Eingebundenseins, die auch für die Aufgabenbestimmung von Medizin und Gesundheitsversorgung für ältere Menschen von Belang sind (Pfaller und Schweda 2019; Ehni et al. 2018). Darüber hinaus kann das höhere Alter aber auch spezifische Dimensionen der Werterfahrung eröffnen. In klassischen Theorien des Guten wird ein gelingendes Leben etwa mit der Erlangung wesentlicher Einsichten und der Ausprägung entsprechender charakterlicher Einstellungen in Zusammenhang gebracht, die Zeit in Anspruch nehmen und daher erst in einem fortgeschrittenen Alter zu erreichen sind (Anton 2016). Neuere gerontologische Theorien wie die der Generativität (Kotre 1984) oder der Gerotranszendenz (Tornstam 2005) greifen solche Vorstellungen auf und suchen sie in einem zeitgemäßen theoretischen Bezugsrahmen neu zu formulieren. Auch und gerade das höhere Alter eröffnet dem Individuum demnach durch Herauslösung aus praktischen Zweckbezügen und Einrücken in umfassendere generationelle, kosmische oder spirituelle Zusammenhänge wichtige Sinnhorizonte, Entfaltungsperspektiven und Erfüllungsmöglichkeiten.

### Die Zeitstruktur guten Lebens und Gesundheitsversorgung im Alter

Um eine differenziertere Sicht auf die Bedeutung der Zeitstruktur guten Lebens für Medizin und Gesundheitsversorgung im Alter zu entwickeln, sind auch verschiedene Dimensionen von Zeitlichkeit auseinanderzuhalten. Dabei lassen sich drei Ebenen zeitlicher Strukturiertheit unterscheiden: (1) grundlegende Koordinaten und Parameter der menschlichen Existenz in der Zeit wie Endlichkeit, Prozessualität und Irreversibilität, (2) durch soziokulturelle Vorgaben geprägte Modelle der zeitlichen Verlaufsstruktur und Stufenfolge des Lebens und (3) die individuelle Lebensgeschichte in ihrer unverwechselbaren biographischen Verlaufsgestalt (Schweda 2020).

Was die *grundlegenden Koordinaten und Parameter der menschlichen Existenz in der Zeit* angeht, so spielt gerade der Aspekt der Endlichkeit als Sterblichkeit eine maßgebliche Rolle für medizinische wie auch pflegerische Entscheidungen im höheren Alter. Je nachdem, ob diese Endlichkeit vorrangig als grundlegende Voraussetzung oder wesentliches Hindernis eines guten Lebens aufgefasst wird, ergeben sich hier andere Bewertungsperspektiven. Im ersten Fall erscheint die Anerkennung und Annahme von Sterben und Tod als wichtiges Anliegen, was auch Konsequenzen für das Angebot und die Inanspruchnahme medizinischer Leistungen haben kann. Das gilt etwa für die Verständigung über angemessene Behandlungsziele im höheren Lebensalter (Callahan 1994) sowie die Beschäftigung mit gesundheitlichen Vorausverfügungen für das Lebensende (Stange und Schweda 2022). Hier lässt eine Akzeptanz der eigenen Endlichkeit, z. B. im Sinne eines natürlichen Zyklus des individuellen und gesellschaftlichen Lebensprozesses, eher Zurückhaltung gegenüber extensiven medizinischen Interventionen sowie den Wunsch nach Begrenzung lebenserhaltender oder -verlängernder Maßnahmen sinnvoll erscheinen (Callahan 1977). Wenn Endlichkeit dagegen vorrangig als ultimative Limitierung aller positiven Perspektiven hedonistischen Lustgewinns oder subjektiver Wunschverfolgung begriffen wird, mag eine extensivere Nutzung medizinischer Möglichkeiten auch im vorgerückten Alter angemessen oder gar geboten erscheinen (Bozzaro 2022).

Auf Ebene der *Verlaufsstruktur und Stufenfolge des Lebens* ergeben sich ebenfalls entscheidende Gesichtspunkte für Gesundheitsversorgung im Alter. Dabei kommt über den Aspekt der Dauer hinaus auch die zeitliche „Binnengliederung“ des Lebens zum Tragen. So entwerfen historisch gewachsene und kulturell geprägte Verlaufsmodelle eine Abfolge von Phasen, Stadien oder Stufen, die jeweils mit einem bestimmten sozialen Status sowie spezifischen Rollen, Handlungsmöglichkeiten und Verhaltenserwartungen verknüpft sind. Sie zeichnen damit eine Art Fahrplan für die Lebensstrecke vor, der Maßstäbe für angemessene oder verfrühte bzw. verspätete Zeitpunkte für medizinische Behandlung und pflegerische Unterstützung nahelegen kann (Schweda 2017b). Hier ist etwa an Bilder zu denken, die dem höheren Alter einen bestimmten Stellenwert im Ganzen des Lebensverlaufs zuweisen, z. B. die Vorstellung eines Lebensabends, einer Zeit der Reife oder aber einer Rückkehr in die Kindheit (Schweda 2020). Daneben sind auch Untergliederungen des späteren Lebens selbst von Belang, etwa die gerontologische Unterscheidung zwischen einem durch anhaltende Funktionalität und Leistungsfähigkeit gekennzeichneten dritten und einem von wachsender Gebrechlichkeit und Hilfsbedürftigkeit geprägten vierten Alter (Wahl und Ehni 2020). Solche Vorstellungen können Angebot und Inanspruchnahme von Gesundheitsversorgung beeinflussen, indem sie im Sinne subjektiver Theorien guten Lebens Erwartungshorizonte sinnvoller und angemessener Vorhaben und Zielsetzungen vorzeichnen. So spielen sie in die Bewertung reproduktionsmedizinischer Möglichkeiten im fortgeschrittenen Lebensalter hinein (s. King et al. in diesem Heft). Darüber hinaus prägen sie auch Annahmen zur Wahrscheinlichkeit und Akzeptabilität gesundheitlicher Beeinträchtigungen im Spannungsfeld von Bagatellisierung oder Pathologisierung. Verbreitet ist etwa die Vorstellung, gewisse körperliche oder geistige Einschränkungen seien im höheren Lebensalter eher hinzunehmen als in jungen Jahren, wobei nicht selten stereotype defizitorientierte Altersbilder im Spiel sind, die einer kritischen Reflexion bedürfen (Beyer et al. 2017). Das betrifft z. B. die Einschätzung des Stellenwertes und angemessenen Ausmaßes beruflicher, sportlicher oder sexueller Aktivität im fortgeschrittenen Alter sowie in der Folge die Beurteilung der Behandlungsbedürftigkeit von Erkrankungen und Beeinträchtigungen wie etwa Schwerhörigkeit, Gelenkarthrose oder erektiler Dysfunktion (zum Überblick Schäfer 2022).

Auch die Ebene der *individuellen Lebensgeschichte* hat wichtige Implikationen für Gesundheitsversorgung im Alter. So wird in der philosophischen Auseinandersetzung mit der Zeitgestalt guten Lebens etwa die Frage erörtert, inwiefern nicht nur die Menge und Dauer beglückender, erfüllender oder wertvoller Ereignisse, sondern auch ihre konkrete Reihenfolge und Verknüpfung im zeitlichen Verlauf eines Lebens von Belang für dessen Bewertung sind (Dorsey 2015). Eine solche Perspektive unterstreicht zunächst die über rein quantitative hedonistische Bilanzen hinausgehende Bedeutung der singulären Projektstruktur und „Dramaturgie“ des individuellen Lebens. Dieses geht nie in allgemeinen existenziellen Koordinaten und soziokulturellen Stufenmodellen auf, sondern hinterlässt stets eine ganz eigene, von persönlichen Plänen und Entscheidungen wie auch von äußeren Umständen und Widerfahrnissen beeinflusste Spur. Welche medizinischen und pflegerischen Entscheidungen angemessen sind, lässt sich demzufolge nicht aus dem chronologischen Alter einer Person ableiten, sondern muss stets unter Berücksichtigung des konkreten biographischen Zusammenhangs ihrer individuellen Lebensgeschichte erörtert werden. In einem Leben, das sich bereits zu einem sinnvollen Ganzen gerundet hat, mag eine Begrenzung medizinischer Maßnahmen annehmbarer erscheinen als in einem in wesentlichen Hinsichten noch „unvollendeten“ Leben (Fuchs 2012). Dabei unterstreicht diese individuelle Zeitdimension auch die Kontingenz und Unabsehbarkeit des konkreten Verlaufs des individuellen Lebens, was z. B. für die Einschätzung der Trageweite gesundheitlicher Vorausverfügungen relevant ist. Schließlich erschließt sich von hier aus auch die Bedeutung narrativer Perspektiven für die ethische Auseinandersetzung mit Gesundheitsversorgung im Alter. Der Sinnzusammenhang des individuellen Lebens ist nie einfach objektiv gegeben, sondern muss erzählerisch hergestellt werden. Gerade im fortgeschrittenen Alter kann das Bestreben, der eigenen Lebensgeschichte im Rückblick eine kohärente narrative Struktur zu verleihen, auch einen maßgeblichen Einfluss auf das Wohlergehen, die Sicht des Lebensendes und damit auch auf die Bewertung medizinischer oder pflegerischer Entscheidungen haben (Streeck 2018).

## Schluss und Ausblick

Im Zeichen steigender Lebenserwartung, gesellschaftlichen Wandels und neuer medizinischer Möglichkeiten geraten hergebrachte Sichtweisen auf das Altern und angemessene bzw. sinnvolle Behandlungsperspektiven im höheren Lebensalter in Bewegung. Es eröffnet sich ein Spannungsfeld zwischen Bagatellisierung und Pathologisierung von Alterungsprozessen, das der Diskussion bedarf. Dabei sind empirisch zu erforschende biologische, psychologische und soziokulturelle Bedingungen des Alter(n)s allerdings mit bislang wenig beachteten strebensethischen Fragen bezüglich der zeitlichen Struktur eines guten Lebens verwoben.

Der vorliegende Beitrag sucht Perspektiven einer ethischen Auseinandersetzung mit derartigen Fragestellungen zu umreißen. Dazu werden philosophische Theorien guten Lebens mit Ebenen menschlicher Zeitlichkeit verschränkt. Das Ergebnis ist ein begrifflich-theoretisches Grundgerüst, in dessen Rahmen sich medizinisch relevante Vorstellungen des Alterns unter ethischen Gesichtspunkten einordnen und erörtern lassen. Besondere Bedeutung kommt dabei der strebensethischen Deutung von Endlichkeitserfahrungen, Lebensverlaufsmodellen und biographischen Narrativen zu, die die Beurteilung der Sinnhaftigkeit und Akzeptabilität von Medizin und Gesundheitsversorgung im höheren Lebensalter auf vielfältige Weisen beeinflussen.

Freilich bedarf das umrissene Gerüst der weiteren konzeptionellen Differenzierung. So ließe sich etwa eine systematisierende Einteilung unterschiedlicher Stufenmodelle und Erzählungsmuster im Hinblick auf ihre ethischen Implikationen entwickeln (Schweda 2020). Darüber hinaus ist die begrifflich-theoretische Perspektive allerdings auch mit der Vielfalt unterschiedlicher lebensweltlicher Vorstellungen guten Alterns im Horizont medizinischer Möglichkeiten zu konfrontieren. Hierzu sind empirische Untersuchungen zu den Sichtweisen älterer Menschen sowie medizinischen und pflegerischen Fachpersonals notwendig, die Aspekte von Alter, Geschlecht, kulturellem Hintergrund sowie sozioökonomischer Schichtzugehörigkeit berücksichtigen. Nur auf diese empirisch informierte und soziokulturell sensibilisierte Weise lassen sich Perspektiven guten Lebens im höheren Alter im Rahmen moderner pluralistischer Gesellschaften medizinethisch fruchtbar machen.
